# Systemic B-cell lymphoma with preceding myelin oligodendrocyte glycoprotein antibody-associated disease: a case report and literature review

**DOI:** 10.3389/fimmu.2026.1675512

**Published:** 2026-02-10

**Authors:** Jing Du, Lei Cao, Xiaokun Qi, Shugang Cao

**Affiliations:** Department of Neurology, Second Affiliated Hospital of Anhui Medical University, Hefei, China

**Keywords:** myelinating oligodendrocyte glycoprotein antibody-associated disease, lymphoma, sentinel lesion, immunophenotyping, biopsy

## Abstract

**Background:**

The co-occurrence of myelin oligodendrocyte glycoprotein antibody-associated disease (MOGAD) and hematological malignancies is infrequently described. We report a rare case of histopathologically confirmed MOGAD complicated by pancytopenia following corticosteroid therapy, which ultimately unveiled a concurrent systemic B-cell lymphoma.

**Case presentation:**

A 76-year-old female presented with bilateral lower limb weakness and numbness persisting for two months. She was initially diagnosed with acute myelitis at an external institution and treated with intravenous methylprednisolone (IVMP). A relapse occurred one month after corticosteroid cessation. Upon admission, magnetic resonance imaging (MRI) demonstrated an enlarging spinal cord lesion and extensive enhancement of the cauda equina nerve roots. A spinal cord biopsy revealed necrosis, chronic inflammatory infiltration, and demyelination, without definitive evidence of tumor cells or atypical lymphoid cells. Immunohistochemical staining revealed a loss of MOG expression, while neurofilament (NF) staining confirmed relative axonal integrity with only focal loss within the affected area. The diagnosis of MOGAD was confirmed via cell-based assays, which showed MOG-IgG positivity in both serum (1:32) and cerebrospinal fluid (1:1). However, the patient was refractory to further IVMP treatment and subsequently developed fever and pancytopenia. Peripheral blood immunophenotyping and bone marrow biopsy eventually established a diagnosis of systemic B-cell lymphoma.

**Discussion:**

The coexistence of MOGAD and systemic B-cell lymphoma is rare. This case suggests that MOGAD may serve as a sentinel paraneoplastic manifestation of an underlying lymphoma.

## Introduction

Myelin oligodendrocyte glycoprotein antibody-associated disease (MOGAD) represents a distinct spectrum of immune-mediated inflammatory demyelinating disorders of the central nervous system (CNS), characterized by a broad spectrum of clinical phenotypes affecting the optic nerves, spinal cord, and brain. In accordance with the 2023 International MOGAD Diagnostic Criteria, the detection of MOG-IgG via cell-based assays (CBA) serves as the obligatory cornerstone for diagnosis (1). While acute episodes generally elicit a favorable initial response to corticosteroids, a subset of patients follows a relapsing course necessitating long-term maintenance immunotherapy.

Although MOGAD is primarily considered a primary autoimmune entity, emerging literature has documented co-occurrences between MOGAD and various hematological malignancies. Specifically, several reports have described patients initially diagnosed with MOGAD who were subsequently found to have concurrent primary central nervous system lymphoma (PCNSL) (2–4). In contrast to these CNS-localized malignancies, its associations with systemic hematological disorders are increasingly recognized, including leukemia (5) and systemic T-cell lymphoma (6). However, the potential pathophysiological link between these disparate conditions—whether restricted to the CNS or manifesting systemically—remains poorly understood. Herein, we present a case initially diagnosed as MOGAD based on clinical, serological, and radiographic findings, the clinical course of which was subsequently complicated by rapid progression to pancytopenia, leading to a confirmed diagnosis of concurrent systemic B-cell lymphoma.

## Case presentation

A 76-year-old female presented to our institution with a two-month history of bilateral lower limb weakness and paresthesia. She was initially evaluated at a referring hospital and diagnosed with acute myelitis, supported by spinal magnetic resonance imaging (MRI) findings of T2-hyperintense signal abnormalities and gadolinium enhancement localized to the conus medullaris (**Supplementary Figure 1**). Following intravenous methylprednisolone (IVMP) pulse therapy, she regained the ability to ambulate with assistance. The patient was discharged on maintenance oral prednisolone (50 mg/day); however, she unilaterally ceased the medication two weeks later. This resulted in rapid clinical deterioration, leading to complete paraplegia and neurogenic sphincter dysfunction. Upon admission to our center, neurological examination revealed severe lower limb paraparesis (Medical Research Council [MRC] scale (7) Grade 1/5) and a sensory level corresponding to the L1 dermatome, with absent Babinski signs.

**Figure 1:**
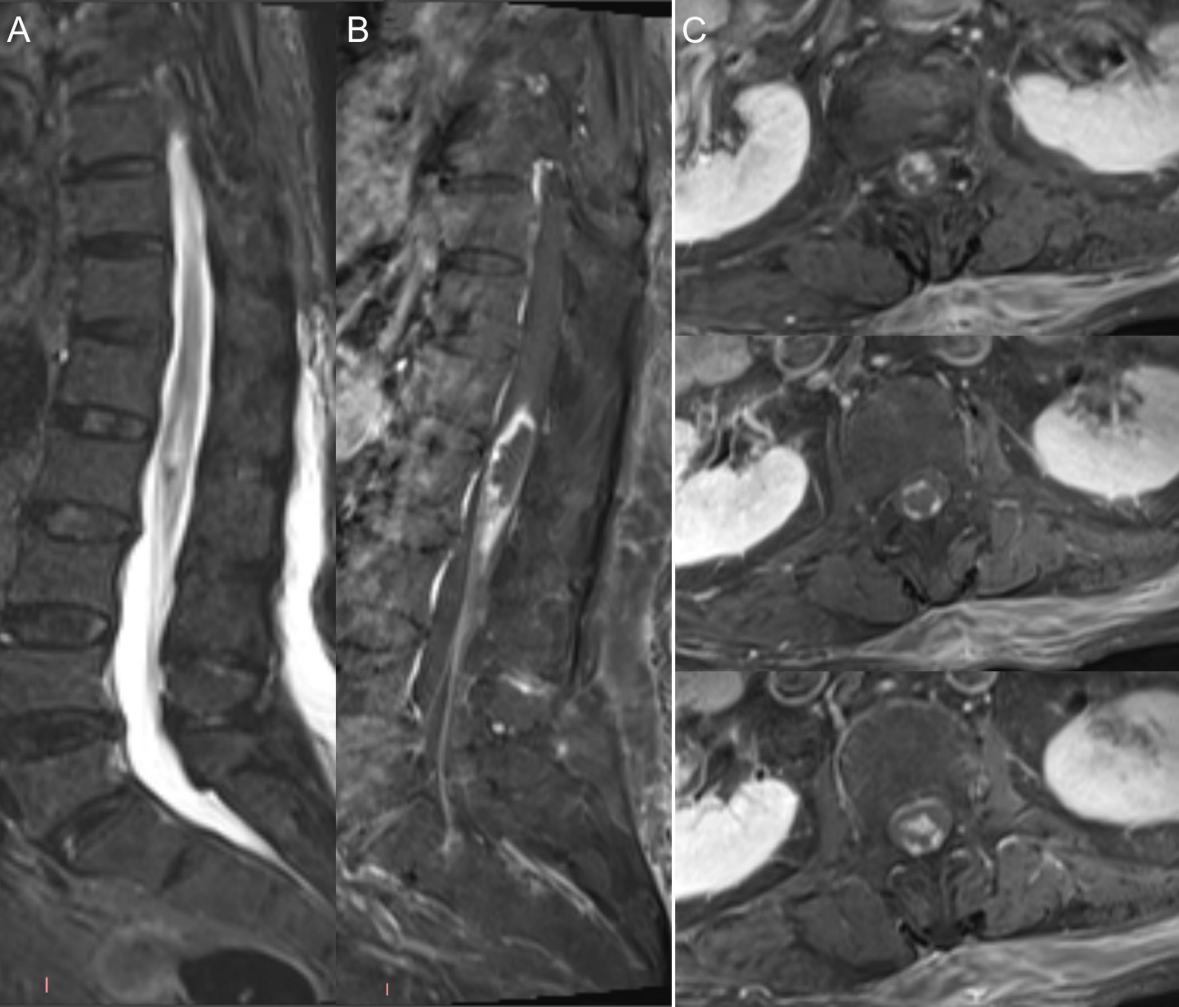
Spinal MRI findings demonstrating disease progression. Spinal MRI demonstrated an enlargement and enhancement of the spinal cord lesion, accompanied by extensive enhancement of the cauda equina nerve roots [**(A)** Sagittal T2WI; **(B)** Sagittal contrast-enhanced T_1_WI; **(C)** Transverse contrast-enhanced T_1_WI.

**Figure 2:**
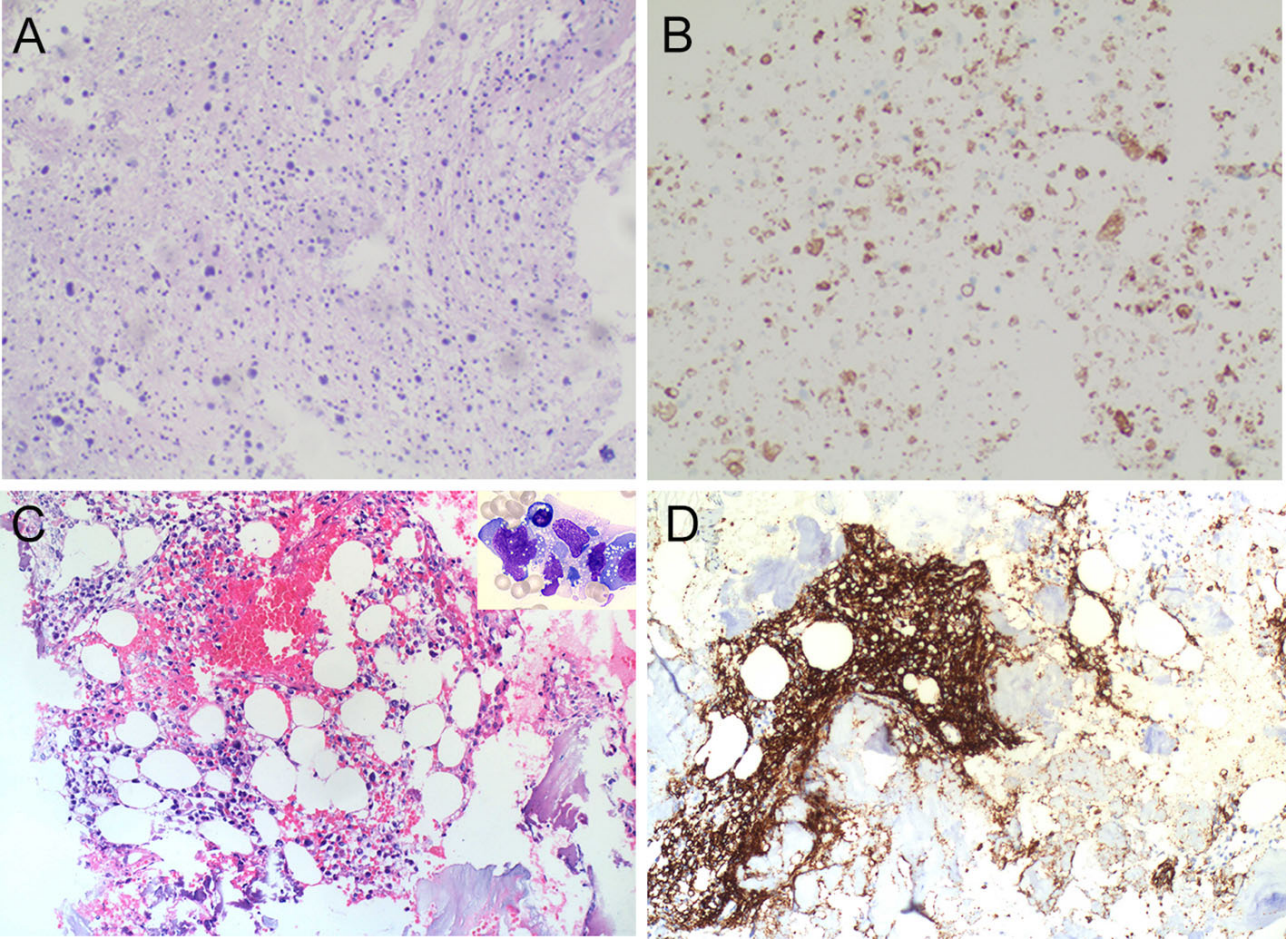
Histopathological findings of the spinal cord and bone marrow. Spinal cord biopsy. Hematoxylin-eosin (H&E) staining shows extensive necrosis, infiltration of chronic inflammatory cells, and numerous foamy macrophages within the interstitium; no neoplastic cells are observed **(A)**. Immunohistochemical staining reveals a loss of MOG expression **(B)**. Bone marrow biopsy. H&E staining demonstrates diffuse proliferation of atypical lymphoid cells in the intertrabecular spaces (occupying ~40% of residual hematopoietic tissue), with suppressed granulopoiesis and erythropoiesis. Significant fibrosis, stromal hemorrhage, and edema are noted in focal areas **(C)** (200×); The upper right corner shows a representative cytomorphology photograph displaying atypical lymphoma cells (Wright-Giemsa stain, 1000×). Immunohistochemical staining for CD20 confirms the B-cell origin of the atypical lymphocytes **(D)** (200×), alongside expression of CD19, PAX-5, and CD79a.

Initial laboratory evaluations, including complete blood count, biochemistry, autoimmune panels (ANA, ANCA, etc.), and tumor markers, were within normal limits. Cerebrospinal fluid (CSF) analysis demonstrated elevated intracranial pressure (210 mmH_2_O), mononuclear-predominant pleocytosis (16 × 10^6/L; reference range: 0-8 × 10^6/L), and increased protein levels (1408 mg/L; reference range: 150–450 mg/L). No atypical lymphoid cells were identified. Notably, CMV-IgG titers were significantly elevated in the CSF (>1000 AU/mL), while other viral markers remained negative. CSF immunoglobulin analysis revealed elevated levels of IgA (15.2 mg/L; reference range: 0–6 mg/L), IgG (239 mg/L; reference range: 10–40 mg/L), and IgM (14.8 mg/L; reference range: 0–13 mg/L). Brain MRI showed no intracranial mass lesions. Conversely, spinal MRI demonstrated lesion expansion with extensive enhancement of the cauda equina nerve roots, raising suspicion of a neoplastic process (**Figure 1**). A subsequent spinal cord biopsy revealed extensive necrotic material containing homogeneous blue-stained spherical vesicles, interstitial chronic inflammatory cell infiltration, and multiple foamy macrophages, with no definitive evidence of tumor cells or atypical lymphoid cells. Immunohistochemical staining revealed a loss of MOG expression, while neurofilament (NF) staining confirmed relative axonal integrity with only focal loss within the affected area (**Figures 2A, B**). Serum and CSF testing via CBA confirmed the presence of MOG-IgG antibodies both in serum (1:32) and CSF (1:1), while AQP4-IgG and GFAP-IgG were negative. Oligoclonal bands were absent in both serum and CSF. Regarding the differential diagnosis of CNS lymphoma, although EBER *in situ* hybridization (ISH) was not performed, the integrated morphological and immunohistochemical profile was highly consistent with MOGAD. Accordingly, the patient was treated with IVMP, initiated at 1000 mg/day for 3 days, followed by a tapering regimen (500 mg, 240 mg, and 120 mg/day, each for 3 days), and transitioned to oral prednisolone (50 mg/day, tapered gradually). At discharge, the patient exhibited neurological improvement, with lower limb muscle strength recovering to MRC Grade 2/5.

Two weeks later, the patient developed fevers accompanied by a productive cough for six days, with a peak recorded temperature of 39.5 °C. The fever persisted despite administration of antipyretics. Laboratory investigations revealed severe pancytopenia, with a nadir white blood cell count of 0.69 × 10^9/L (neutrophils 10.5%, lymphocytes 69.0%), red blood cell count of 2.28 × 10^12/L, hemoglobin of 70 g/L, and a platelet count of 39 × 10^9/L. Further diagnostic workup for the cytopenia indicated markedly elevated soluble interleukin-2 receptor (sIL-2R/sCD25) levels (6434.8 pg/mL; reference: 0–600 pg/mL) and serum ferritin (18,100 µg/L; reference: 20–290 µg/L), alongside hypofibrinogenemia (0.84 g/L; reference: 1.7–4.0 g/L).

The patient was transferred to the Hematology Unit for further evaluation. Peripheral blood immunophenotyping identified two distinct but phenotypically identical B-lymphocyte populations (totaling 8.7% of nucleated cells) exhibiting a CD5−CD19+CD20dimCD22+CD23+CD10−CD38− profile, with a matching CD20 mean fluorescence intensity (MFI) of 4.85 and a k/λ light chain ratio of 1.60. Bone marrow aspirate demonstrated infiltration by abnormal lymphocytes (approximately 29%), while biopsy confirmed a diagnosis of aggressive B-cell lymphoma associated with secondary hemophagocytic lymphohistiocytosis (**Figures 2C, D**). Consequently, a definitive diagnosis of concurrent MOGAD and Ann Arbor stage IV B-cell lymphoma was established. Following adequate infection control, the patient was initiated on the R-CDOP regimen (rituximab, cyclophosphamide, doxorubicin, vincristine, and prednisone). However, given the poor prognosis and deteriorating clinical status, the patient’s family elected for palliative care. The patient succumbed to the disease shortly after discharge.

## Discussion

MOGAD is a distinct CNS inflammatory demyelinating disorder characterized by a broad clinical spectrum (1). Conus medullaris involvement represents a relatively infrequent but highly characteristic feature of this condition. In the present case, the patient initially presented with neurological deficits localized to the conus medullaris. Although IVMP treatment at a referring institution yielded partial clinical improvement, her symptoms relapsed during steroid tapering. Contrast-enhanced MRI revealed an enlarging spinal cord lesion with prominent enhancement of the cauda equina, raising suspicion for a neoplastic process. However, spinal cord biopsy demonstrated extensive necrosis with chronic inflammatory cell infiltration; notably, immunohistochemical analysis confirmed loss of MOG immunoreactivity in the absence of neoplastic lymphoid infiltration. While MOGAD is typically characterized by axonal preservation, the focal axonal loss noted here may be attributed to the severe necrotic changes and the subacute-to-chronic disease progression at the time of biopsy. This degree of axonal damage correlates with the clinically aggressive and destructive course. The diagnosis of MOGAD was subsequently confirmed by MOG-IgG seropositivity in both serum and CSF via CBA. Unexpectedly, the patient developed a fever two weeks later, coinciding with the emergence of pancytopenia. A diagnosis of systemic B-cell lymphoma was ultimately established via peripheral blood immunophenotyping and bone marrow biopsy.

It is clinically imperative to distinguish our case from cases of PCNSL, as the clinical course and management differ significantly. Historically, “sentinel lesions”—precursor inflammatory demyelinating episodes—have been predominantly associated with PCNSL rather than systemic malignancies. For instance, Bajagain et al. (8) analyzed 12 cases of PCNSL preceded by such sentinel lesions. The majority (7/12) were initially diagnosed as multiple sclerosis, followed by unspecified demyelination, and only one case met the criteria for MOGAD (4). To our knowledge, only three instances of MOGAD complicated by PCNSL have been reported to date (2–4). A common feature shared by two of these patients was the presence of intracranial lesions, where initial biopsies suggested MOGAD, but subsequent rapid clinical deterioration led to a confirmed PCNSL diagnosis via repeat biopsy or CSF analysis (2, 3). In contrast, our patient’s malignancy was entirely systemic at presentation; extensive imaging and initial spinal cord biopsy provided no evidence of PCNSL or CNS involvement by the systemic lymphoma.

The pathophysiology of sentinel lesions in the context of lymphoma remains elusive. Uzura et al. (2) reported a case of PCNSL concurrent with MOGAD in which MOG-IgG titers remained elevated despite chemotherapy, suggesting that MOG-IgG antibodies are not directly produced by tumor B-cells and that the relationship is likely non-causal (2, 9). In our case of systemic B-cell lymphoma, the absence of tumor cells in the spinal cord biopsy suggests that the MOGAD-like presentation may represent a paraneoplastic-like immune response. While sentinel lesions are a recognized phenomenon in PCNSL, our case highlights that they can also occur in systemic B-cell lymphoma. We hypothesize that the sentinel lesion resulted from an early systemic immune response against the occult lymphoma, leading to secondary exposure of MOG antigens to the immune system, possibly through transient inflammation-induced blood-brain barrier disruption (2).

In conclusion, while MOGAD is more commonly discussed as a potential sentinel manifestation of PCNSL, its co-occurrence with systemic B-cell lymphoma is exceptionally rare. In patients with demyelinating lesions who exhibit a suboptimal response to conventional steroid therapy or develop systemic symptoms like pancytopenia and fever, clinicians should rigorously investigate the possibility of occult systemic malignancy. This case underscores that MOGAD can serve as a sentinel manifestation for lymphomas beyond the CNS.

## Data Availability

The original contributions presented in the study are included in the article/supplementary material. Further inquiries can be directed to the corresponding author.

## Supplementary material

Supplementary Figure 1 Initial spinal MRI presentation at an external institution. Sagittal T_2_WI demonstrates a hyperintense signal abnormality localized to the conus medullaris (**A**). Contrast-enhanced T_1_WI in sagittal (**B**), coronal (**C**), and axial (**D**) planes reveals significant enhancement of the lesion.
