# Supplementary figures and images for: Systemic B-cell lymphoma with preceding myelin oligodendrocyte glycoprotein antibody-associated disease: a case report and literature review
Source: Front Immunol. 2026 Feb 10;17:1675512. doi: 10.3389/fimmu.2026.1675512 (PMC12929389; doi:10.3389/fimmu.2026.1675512)

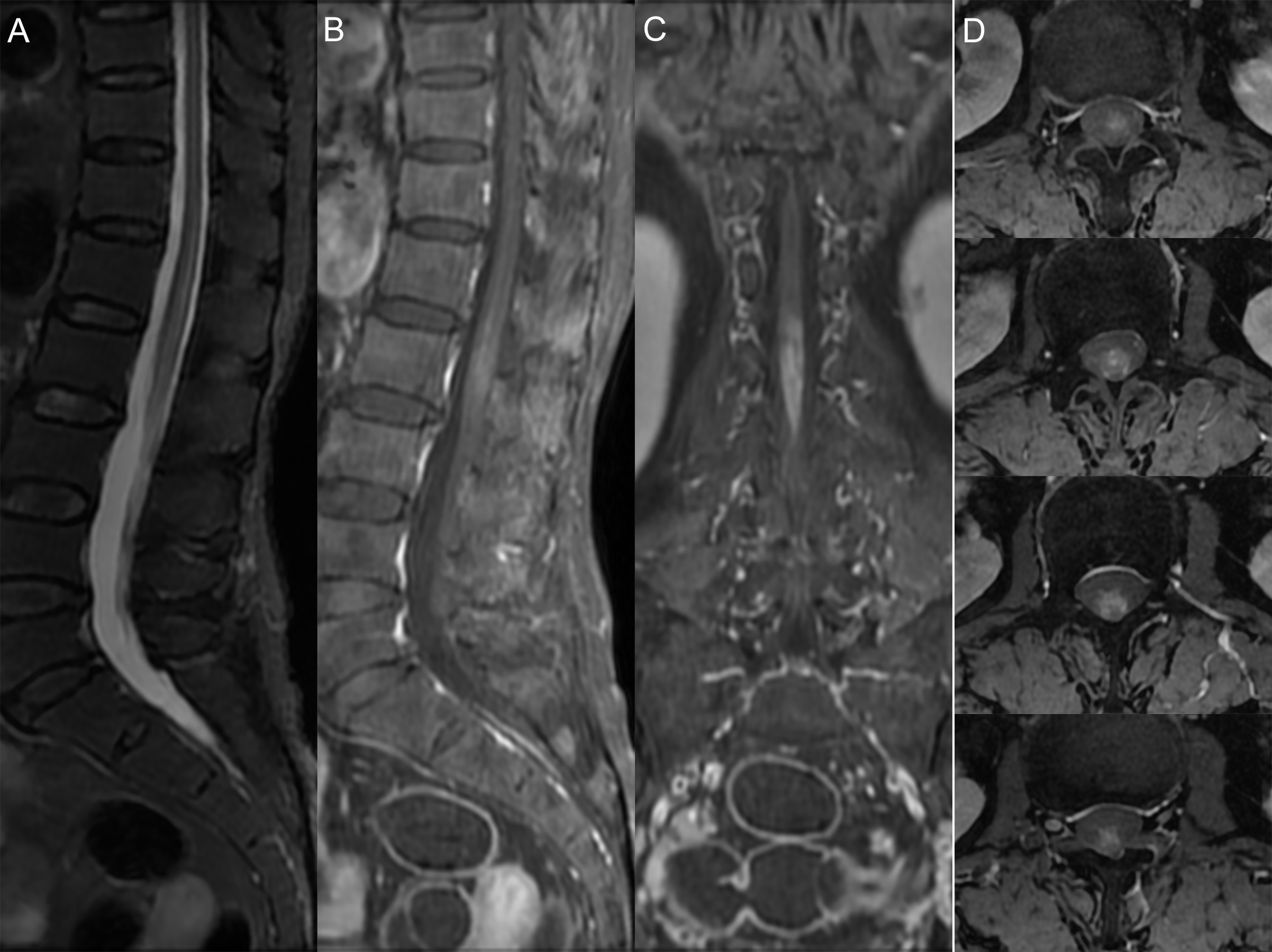

Supplement: Supplementary Figure 1 — Initial spinal MRI presentation at an external institution. Sagittal T2WI demonstrates a hyperintense signal abnormality localized to the conus medullaris (A). Contrast-enhanced T1WI in sagittal (B), coronal (C), and axial (D) planes reveals significant enhancement of the lesion. [file Image1.tif]
